# Managing Complexity in Socio-Technical Systems by Mimicking Emergent Simplicities in Nature: A Brief Communication

**DOI:** 10.3390/biomimetics9060322

**Published:** 2024-05-28

**Authors:** Andrea Falegnami, Andrea Tomassi, Giuseppe Corbelli, Elpidio Romano

**Affiliations:** Faculty of Management Engineering, International Telematic University Uninettuno, 00186 Roma, Italy; andrea.tomassi@uninettunouniversity.net (A.T.); giuseppe.corbelli@uninettunouniversity.net (G.C.); elpidio.romano@uninettunouniversity.net (E.R.)

**Keywords:** complex adaptive systems, complexity, emergentism, neurosciences, reductionism, resilience engineering, simplicity

## Abstract

In the context of socio-technical systems, traditional engineering approaches are inadequate, calling for a fundamental change in perspective. A different approach encourages viewing socio-technical systems as complex living entities rather than through a simplistic lens, which enhances our understanding of their dynamics. However, these systems are designed to facilitate human activities, and the goal is not only to comprehend how they operate but also to guide their function. Currently, we lack the appropriate terminology. Hence, we introduce two principal concepts, simplexity and complixity, drawing inspiration from how nature conceals intricate mechanisms beneath straightforward, user-friendly interfaces.

## 1. Introduction

Socio-technical systems, characterized by the intricate interplay between human activities and technological infrastructure, represent a concept that is becoming increasingly central in engineering studies concerning human activities [1]. Indeed, some scholars state that the unique characteristics of humans as a species is the ability to design and produce artifacts to enhance their capabilities [2]. Mankind would not be sapiens if it had not been faber before [3]. Virtually no activity exists that does not involve some artifact, albeit nonmaterial. Traditional engineering, adhering to the Cartesian–Newtonian reductionist paradigm, designs and manages “technical” systems by decomposing phenomena into simpler elements—a method foundational to scientific inquiry that has yielded significant insights in fields where phenomena can be isolated for analysis [4]. However, this approach tends to miss emergent behaviors and nonlinear interactions in complex systems, which are essential for their understanding, design, and management. The traditional reductionist approach, characterized by the pursuit of simplicity through decomposition, is increasingly proving inadequate for systems that are deeply intertwined with human activities, as socio-technical systems are inherently complex [5]. The concern is not only that this “simplistic” view does not provide a satisfactory explanation of the facts, but also that it has led to errors, sometimes fatal [6]. Since the late 20th century, recognizing and managing the complexity of socio-technical systems has gained importance. The perspective of viewing organizational systems as biological systems has been explored in some works [7]. More recent methodologies like resilience engineering, viewing these as complex adaptive systems, advocate for a shift towards holistic and non-reductionist systemic approaches [8].

Complex adaptive systems necessitate a focus on emergent phenomena–complex behaviors and properties stemming from interactions among subsystems that cannot be foreseen by examining components in isolation. Emergentism (also known as holism or contextualism) offers a perspective that acknowledges and engages with this inherent complexity. The shift towards acknowledging emergent behaviors has been embraced by resilience engineering, leading to significant insights into the study of socio-technical systems, where such behaviors are crucial for functionality and resilience. This perspective has been effectively applied in analyzing organizations and critical environments like surgical suites, air traffic control, nuclear plants, and construction sites [6,9,10,11,12].

Complexity studies have been instrumental in shedding light on the dynamics of socio-technical systems, offering insights that transcend traditional disciplinary boundaries, but the time has come to add design and synthesis to description and analysis. It is time to move from science—which deals with what is—to technology—which deals with what should be; it is time to bring the design dimension of engineering into the complexity studies discourse [13].

Recognizing the need to integrate technological infrastructure with social, cultural, and procedural dynamics, it is imperative to develop organizations that balance both technological proficiency and human needs, creating resilient, adaptive environments for long-term success. However, a challenge remains in the inadequate vocabulary to fully capture this design dimension. This communication aims to share insights from extensive research on emergent simplicity in complex adaptive systems, conducted by a multidisciplinary team, drawing valuable comparisons between engineering, biology, and cognitive science.

## 2. The Need for a New Lexicon

This communication aims to spread insights into socio-technical systems, emphasizing the importance of frameworks like material engagement theory, enactivism, and theories of situated and distributed cognition [14,15,16]. Highlighting humans as natural cyborgs (Homo faber), it underscores that organizations cannot operate without both conceptual and material artifacts; this is intrinsic to their functionality. We should conceptualize socio-technical systems as living organisms that interact with their surroundings by creating their organs (or tissues, or proteins), aspiring for their collective behavior to mimic that of an amoeba, reflecting adaptability and integration with the environment.

While the essence of engineering is to develop usable and effective artifacts that meet project requirements, we have incorporated complexity into our systems, mirroring the complexity found in nature, but have yet to achieve the simplicity of action that nature exemplifies. Nature abounds with instances of simplicity emerging from complex systems, serving as a reminder that our engineering efforts should strive towards simplifying actions within these intricate systems. The introduction of a new lexicon is necessary, as it serves as a foundational artifact to manipulate and articulate these subsequent emerging concepts effectively among different kinds of engineers.

The hunt of a predator, such as a wolf tracking its prey, serves as a metaphor for understanding complexity and simplicity within systems. This act involves a multitude of simultaneous mechanisms: lessons learned from parental guidance, instinctual behaviors honed through evolution, cardiovascular homeostasis, and metabolic cellular functioning. Yet, when observed from a certain distance, these intricate processes coalesce into what appears as simple behavior, which can be analyzed through ethological or ecological lenses. This simplicity, on one hand, conceals all the phenomena just mentioned, and on the other, presupposes them all, since without this entire hierarchy of complexity, such a simple interpretation would not be possible. A similar dynamic unfolds in socio-technical systems, where technology builds upon a hierarchy of other technologies, illustrating how complex systems underpin and facilitate seemingly straightforward functionalities.

The challenge in system design is to conceal complexities without sacrificing functionality or user engagement. Achieving a simple user interface that retains the system’s full capabilities demands a balance of design, technology, and understanding of user psychology. Furthermore, the diversity in user competence levels necessitates adaptable interfaces that cater to both novice and expert users, complicating the design process. Finding the right balance between simplicity for ease of use and complexity for full functionality involves iterative design and testing, which often lead to compromises that may not satisfy all user groups equally.

As researchers, we continually recognize simple solutions within natural complexity, which is why we believe that the future of designing and managing socio-technical systems lies in our ability to understand and leverage both the complexity and the emergent simplicities of natural systems. In this sense, the contrast between reductionism and emergentism highlights a fundamental tension in the study of socio-technical systems: the need to navigate between the complexity of these systems and the artifacts’ simplicity required for their operation. Embracing complexity, ultimately, does not mean giving up on simplicity [17].

To realize these artifacts, however, we must first be able to conceptualize them; hence, we need proper semantics, which needs a lexicon to rely on. Such a lexicon could bridge the gap between complexity and simplicity, enabling researchers, practitioners, and policymakers to articulate and address the challenges and opportunities inherent in these systems.

## 3. Explaining the Complexity–Simplicity Dichotomy by Taking Nature as an Example

The dialogue between complexity and simplicity is central to our discourse and represents the foundational core of such semantics.

The interplay between simplicity and complexity is the dynamic that shapes the nature of complex adaptive systems, both natural and artificial. Simplicity, characterized by ease and clarity, arises when complexity is organized and optimized within systems. This occurs, for example, when biological control structures (e.g., nerve ganglia) inhibit certain actions (e.g., the “fight or flight” mechanism), when sense organs filter signals by preventing information overload (e.g., cutoff frequencies in vision and hearing) or when coordinating tissues produce fine-resolution results (e.g., fine muscle movement).

Complexity, on the other hand, refers to the intricate and multifaceted aspects of systems that arise from the interactions among their components. This dynamic is central to the understanding of systems, where simplicity and complexity coexist and can have the power to inform design and expected functionality. In any case, complexity and simplicity are complementary aspects of reality, depending on the perspective from which systems are viewed [18]. In complexity, regular patterns or behaviors continually emerge. It is this emergence that we must be able to design in order to understand and manage complexity effectively. The challenge, then, is not simply to reduce complexity, but to navigate within it, identifying and exploiting emerging simplicities as design principles and operational insights.

## 4. Conclusions

We believe that the entire dynamic interaction between complexity and simplicity can be encapsulated by two new concepts, simplexity and complixity, capable of completing the basic framework for articulating the simple–complex dichotomy.

Simplexity refers to the intrinsic complexity of a system that is artfully masked by an overlay of simplicity. This concept bears witness to nature’s ability to evolve systems that, despite being complex in their internal functioning, present an interface or a model of interaction that is extraordinarily simple to use. This is a concept not new in biology and the study of organizations, illustrating how sophisticated outcomes and behaviors can emerge from the complex interplay of simpler rules and elements [19,20].

In biomimetics, simplexity is reflected in the design of systems and materials that emulate the elegant efficiency of natural processes, where a myriad of microscopic interactions translates into macroscopic phenomena that are easily understandable and interactable by humans, concealing the complexity at an emergent level [21]. For example, the structure of a leaf, with its complex vascular network that efficiently distributes nutrients and water throughout the organism, inspires the design of water distribution networks and cooling systems that maximize efficiency through seemingly simple designs.

Another example of simplexity is provided by the process of cellular autophagy, where cells self-regulate and renew by recycling components [22]. This self-sustaining mechanism ensures vitality and efficiency, offering a metaphor for socio-technical systems’ need to continually evolve by reassessing and refining their components. By biomimetically mimicking autophagy, socio-technical systems can adopt sustainable practices for renewal of functional structures, ensuring they remain dynamic and responsive to the needs of their environment [21].

Complixity, an even newer concept that we believe should find a place in the discourse, delves into the dynamics of simplicity emerging from the system’s behavior when driven by environmental constraints and other exogenous factors. This concept expresses the characteristic simplicity emerging from contextual factors, which is why we prefer the term “contextualism” over “emergentism” in the case of complixity.

Complixity accounts for how systems, influenced by external stimuli, adapt and evolve in a regular manner within the complex environments of which they are part. Given the fractal hierarchical structure of complex systems, at each higher level, the old context is incorporated into what becomes a new higher-level complex system or supersystem. The regularity of contextual actions allows equally regular phenomena to be shaped in response. It represents a different kind of simplicity, achievable only through confrontation with otherness.

The phenomenon of bacterial quorum sensing exemplifies the concept of complixity—complex, decentralized decision-making processes that culminate in a simple, unified outcome [23]. In this biological process, individual bacteria communicate through chemical signals, coordinating their actions based on population density, representing a typical case of a simplicity that has emerged from the interaction of many complexities [24]. This mirrors the challenge socio-technical systems face in facilitating effective communication and decision-making within organizations. By emulating quorum sensing, socio-technical systems can adopt more fluid and adaptive communication strategies, allowing for decentralized decision-making that remains coherent and aligned with organizational goals.

In biomimetics, complixity is reflected in the design of systems and materials that emulate the emergence of new simple solutions obtainable from the juxtaposition and encounter of two ecological, evolutionary, cognitive niches. For example, the encounter with a new host organism allows a bacterium to overflow into an uncharted evolutionary territory. Similarly, a technology that has been well established can be used effectively when combined with newer technologies or applied in fields different from its original ones. This idea underpins the concept of exaptation in design science research and TRIZ (Theory of Inventive Problem Solving) [25,26]. It illustrates how the integration of different systems or ideas can lead to innovative adaptations and solutions, highlighting the potential of complixity to foster creative and adaptive designs that respond to complex challenges by bringing together disparate elements in novel ways.

Both simplexity and complixity support a balanced approach to the design and management of systems, where the elegance of simplicity is achieved without sacrificing the richness of complexity. This balance ensures that systems are neither overly simplified to the point of ineffectiveness nor made unnecessarily complex at the expense of usability and accessibility. In the field of socio-technical system design, these principles encourage the creation of user-centered systems that are easy to understand and interact with, yet powerful and capable of complex operations and adaptations. The applicability of simplexity and complixity extends beyond physical design to include the organizational and procedural aspects of socio-technical systems. Organizations can streamline processes, improve communication, and promote innovative and inclusive cultures. The result is a more cohesive and efficient system that leverages the strengths of its human and technological components. Moreover, simplexity and complixity are two constructs particularly relevant in terms of multiscale methods and the structure–function relationship. Indeed, biomimetics employs multiscale methods to bridge the gap between the microscale behaviors of materials and their macroscale applications [27]. This approach, reminiscent of the principles of scaling and renormalization in physics, reveals how simple behaviors at one scale are the result of complex interactions at a smaller scale.

Understanding the crucial relationship between substructure and function in complex systems underscores the importance of considering the interaction of constituent elements in engineering desired outcomes, akin to what is proposed through the ladder of structural levels suggested by cognitive neuroscience [28]. This perspective illuminates how the simplicity of system functions often emerges from the complexity of interactions among its constituent parts.

The difference between simplexity and complixity can be understood by analyzing their definitions within the context of complexity sciences. In summary, simplexity concerns the emergence of simple behaviors from complex interactions within the same structural context, while complixity concerns the emergence of new forms of simplicity from interactions between different structural contexts. Simplexity refers to the emergence of simple characteristics at a current level of the hierarchy of reality as a direct consequence of the high-intensity, nonlinear interaction of rules. These simple characteristics emerge independently of the complexity and fine structure of the underlying level. In other words, simplexity occurs when sub-structural rules interact within a limited space of combinations, producing behavior that appears simple despite the complexity of the underlying interactions. Complixity, on the other hand, occurs when sub-structural rules belonging to entirely different structural units interact. This type of interaction expands the space of possibilities and allows contact between previously distinct elements, combining them and creating new dynamics of interaction. Complixity thus represents an emergent simplicity in a new context, resulting from the interaction of different structural units and is distinguished from simplexity by its contextual origin and the dynamic of its generation.

Simplexity and complixity can guide us in managing these aspects, offering a window into the soul of nature’s design philosophy, where the complexities of life processes are distilled into principles of elegant simplicity. This biomimetic lens not only advances our ability to develop sustainable, efficient, and adaptable technologies and systems, but also deepens our understanding of the intrinsic simplicity that guides the complexity of the natural world.

The principles of simplicity and complexity inherently require the merging of knowledge from multiple disciplines, including biology, engineering, psychology, and design. Fostering effective interdisciplinary collaboration poses significant challenges due to the different terminologies, methodologies, and goals of these fields. This collaboration is essential to translate complex natural phenomena into simple and applicable design principles that can be universally adopted and implemented.

The present brief communication introduces two constructs that are the outcome of more extensive and rigorous research. The purpose is to expose these conceptual artifacts to academic discussion and debate. We believe that biomimicry can benefit from these concepts and help define new ones in the field of socio-technical systems. The essence of emergentism (contextualism), reflected in the remarkable simplicities discovered amid complexity, further validates the notion that simplicity is not simply the absence of complexity, but its hallmark.

This approach for the design of socio-technical systems is an infant that has just uttered two words: simplexity and complixity. Much more will have to follow if we are to build socio-technical systems as they should be in the future, beyond the mere study of what they are now.

Summarizing in layman terms, simplexity is a unary operator, a filter that omits details; complixity is a binary operator—an AND or an OR—that allows for new solutions through comparison. This concept highlights the adaptability and responsiveness of systems to external challenges, guiding the development of socio-technical systems that can dynamically adapt to environmental changes, user demands, and technological advances.

## Data Availability

No new data were created or analyzed in this study. Data sharing is not applicable to this article.
